# Development and validation of a person-centered antenatal care scale for low- and middle-income countries

**DOI:** 10.21203/rs.3.rs-5597466/v1

**Published:** 2025-05-07

**Authors:** Patience A. Afulani, Hawa Malechi, Daniel Enos Sekwo, Jaffer Okiring, Moro Ali, Osamuedeme J. Odiase, Beryl Ogolla, Joyceline Kinyua, Linnet Ongeri, Raymond A. Aborigo

**Affiliations:** Department of Obstetrics, Gynecology, and Reproductive Sciences, University of California San Francisco; Tamale Teaching Hospital; Navrongo Health Research Centre; Global Programs for Research and Training; Navrongo Health Research Centre; Department of Obstetrics, Gynecology, and Reproductive Sciences, University of California San Francisco; Global Programs for Research and Training; Kenya Medical Research Institute; Kenya Medical Research Institute; Navrongo Health Research Centre

**Keywords:** prenatal/antenatal care, person-centered care, experience of care, quality of care, respectful maternity care, patient-reported experience measure, LMIC, Ghana, Kenya

## Abstract

**Background:**

Person-centered antenatal care (PCANC)—antenatal care that is respectful and responsive to people’s needs, values, and preferences—is essential to achieving optimal pregnancy outcomes. Yet, no validated tools exist to comprehensively measure PCANC in low- and middle-income countries (LMICs). We aim to develop and validate a tool to comprehensively measure PCANC relevant to women’s experiences in LMICs.

**Methods:**

We followed standard procedures for scale development. This included a literature review to adapt items from a prior scale developed in the United States and to generate new items relevant to LMICs; expert reviews with maternal health experts, health care providers, and women with lived experiences (currently pregnant or previously pregnant) to assess content validity; and cognitive interviews and pretesting with pregnant and postpartum women to evaluate clarity, appropriateness, and relevance of the questions. Questions were iteratively revised at each stage and administered in a survey to 300 pregnant (third trimester) and 300 postpartum women (who gave birth within six months of the interview) in the Upper East Region of Ghana. Following data analysis from the first survey, the questions were revised and administered to 2000 postpartum women in Ghana and Kenya (1000 per country). The survey data were used in psychometric analysis to assess construct and criterion validity, and internal consistency reliability.

**Results:**

Iterative exploratory factor analysis was used to reduce the number of items from over 60 to 36. The 36 items load onto one dominant factor, with three factors having eigenvalues greater than one. Items are grouped into three conceptual domains representing subscales for “dignity and respect,” “communication and autonomy,” and “responsive and supportive care.” The Cronbach’s alpha for the full scale is 0.90, and the subscales are each > 0.7. The summative PCANC scores correlate with global measures of antenatal care quality, satisfaction, and future care location, suggesting good criterion validity.

**Conclusions:**

The PCANC scale has high validity and reliability in the sample of prenatal and postpartum women in Ghana and Kenya. This scale will facilitate efforts to measure and improve respectful and responsive antenatal care in LMICs.

## Background

High-quality antenatal care (ANC) is critical for preventing, identifying, and managing pregnancy complications to ensure the health of mothers and their babies[1]. The proportion of women receiving ANC has increased globally, including in low- and middle-income countries (LMICs), with over 80% of women in sub-Saharan Africa (SSA) having at least one ANC contact during their pregnancy[2]. Although current World Health Organization (WHO) guidelines recommend eight antenatal contacts[1], less than two-thirds of women in SSA achieve the previously recommended four visits [2]. Additionally, significant gaps in quality persist, both in service provision and experience of ANC, which contributes further to the low coverage[3–5].

Person-centered care during the pregnancy and childbirth continuum is critical to maternal health from both a quality of care and human rights perspective[6, 7]. We define person-centered antenatal care (PCANC) as ANC that is respectful, compassionate, and responsive to the needs, preferences, and values of pregnant women and gender-diverse people (women used subsequently for brevity), adapted from the definition of person-centered care by the Institute of Medicine[8]. PCANC has both direct and indirect effects on maternal and neonatal outcomes through its effect on patient care, engagement, and health-seeking behavior, including skilled attendance at birth[9, 10]. The WHO recommendations for ANC highlight the importance of a positive experience[1]. Operationalizing these recommendations requires measuring women’s experiences to identify gaps and inform interventions. However, efforts to measure women’s ANC experiences have lagged, with most efforts focused on childbirth[11].

Studies on ANC have primarily focused on frequency and timing[12–17]. Among the few that address quality of care, most focus on the content of ANC service provision, with relatively fewer on the person-centeredness of care [4, 5, 18–22]. This is due to the lack of validated measures for PCANC. A recent scoping review of measures that align with the WHO recommendations for ANC found that existing measures prioritize clinical content of ANC—with measures for women’s experiences of care notably lacking[23]. The few studies that include some measures of ANC experience have, however, highlighted gaps in PCANC. For example, a study in Kenya found that about one-third of women did not often understand the purposes of tests and medicines received and did not feel able to ask questions to the healthcare provider, only half reported being consistently asked if they had any questions, and about a third reported that they could never discuss issues in private[4]. While studies in LMICs have measured various aspects of PCANC, there is still no validated PCANC scale for LMICs.

Similar to ANC, there were few validated quantitative measures of person-centered care during childbirth until recently[24–26]. However, increased interest in women’s experiences during childbirth led to the development of several quantitative tools for this in the last decade[27]. Among the comprehensive validated tools for measuring women’s experiences during childbirth is the Person-centered maternity care (PCMC) scale, initially developed and validated in Kenya and India[26, 28–30]. The PCMC scale, which has domains for communication and autonomy, dignity and respect, and supportive care, has had widespread uptake, with subsequent validation and use in many countries in Africa, Asia, and America[31–36]. The scale has enabled us to extend research on predictors and consequences of PCMC [37–39] and has been used to evaluate interventions to improve PCMC[40, 41]. Furthermore, the PCMC scale informed the development of person-centered family planning and abortion scales[42–44], and notably, a person-centered prenatal (antenatal) care scale, focusing on the experiences of people of color in the United States (the PCPC-US scale)[45].

The PCPC-US scale, which has 34 items with three domains for communication and autonomy, dignity and respect, and responsive and supportive care, was developed in the United States (US) based on standard procedures for scale development but with a focus on the experiences of Black women and other people of color in the US. The psychometric analysis in studies shows that the scale has high validity and reliability in a sample of predominantly Black women [45] and predominantly Latina women in the US [46]. The PCPC-US has, however, not been validated in an LMIC. We seek to address this gap. Our aim in this study is to adapt the PCPC-US scale to make it relevant to LMIC contexts, with an initial validation in Ghana and Kenya. We refer to this scale for LMICs as a PCANC scale (as against PCPC) because the term ‘antenatal care’ is more commonly understood in LMICs than ‘prenatal care,’ although both refer to the same service during pregnancy.

## Methods

We followed standard procedures for scale development as was used in the development of the PCMC and PCPC-US scales to ensure both conceptual and psychometric adequacy[47, 48].

### Setting

The initial adaptation and validation activities took place in Ghana in the Upper East Region (UER). The final confirmation sample is the baseline data for an ongoing trial in northern Ghana’s Upper East and North East Regions and Migori and Homa Bay Counties in western Kenya. These sites have all been previously described[49, 50]. The Upper East and North East Regions are neighboring regions located in the northeastern corner of Ghana, both sharing borders with Togo to the east. The Upper East Region shares boundaries with Burkina Faso to the north and the North East Region to the south. The Upper East region is divided into 15 districts, with 11 district hospitals, 67 health centers, about 419 Community-Based Health Planning and Services (CHPS) compounds, and one regional hospital that serves as a referral center for the district hospitals[51, 52]. The North East is divided into six districts, with five district hospitals, 21 health centers, and 154 CHPS compounds [53].

Migori and Homabay are also neighboring counties along Lake Victoria in western Kenya. They each have eight sub-counties, each with a sub-county hospital and one county referral hospital. There are about 155 and 263 health facilities in Migori and Homa Bay, respectively, including county and sub-county hospitals, health centers, and faith-based and private health facilities[54].

### Procedures to ensure conceptual adequacy

The first step was a **literature reviewon** women’s experiences during ANC in LMICs to update the items in the PCPC-US scale. Although the PCPC-US scale was developed following a comprehensive literature review to define the construct and domains, the focus for the items was on the experiences of people of color in the US. Thus, we reviewed the literature on women’s experiences during ANC in LMICs to identify issues that are most important during ANC to pregnant people in LMICs. This included reviewing the systematic review of what matters to women during ANC, the WHO guidelines on ANC for a positive pregnancy experience, and previous publications on PCANC[1, 55, 4]. Following the literature review, we assessed the PCPC-US scale to identify which items may apply to ANC in LMICs. We included additional relevant items not already captured based on the literature review.

The next phase involved **expert reviews** to assess content validity by asking experts to judge the comprehensiveness of items for measuring a construct and their relevance to the target population[56, 57]. Experts were purposively recruited to include maternal health experts, healthcare workers, and women with lived experiences with pregnancy (currently pregnant or had previously been pregnant). Eleven external experts—three researchers based in Ghana, three healthcare providers, three pregnant women, and two women who had recently given birth (recruited from health facilities in the Upper East region of Ghana)—in addition to the research team (four maternal health researchers, two of whom have clinical experience), participated in the expert reviews. This number exceeded the recommended sample size of at least six expert reviewers[58]. Each reviewer was first sent the initial list of questions to review individually. For each question, they were asked to think about its relevance to measuring PCANC in general (as defined for them) and to the ANC experiences of pregnant women in Ghana to rank it on a scale of 0 to 3 (“0” not relevant, “1” somewhat relevant, “2” quite relevant, and “3 highly relevant”). Experts were also asked to assess the comprehensiveness of the items for measuring PCANC on a scale of 0 to 3 (0. Not comprehensive; 1. Slightly comprehensive; 2. Comprehensive; 3. Very comprehensive) and whether all relevant aspects of PCANC were covered by these items on a scale of 0 to 3 (0. No; 1. Yes, somewhat; 2. Yes, definitely). In addition, they were asked to make recommendations on what to add, remove, or change. Following the individual reviews, experts were brought together as a group for further discussion and consensus-building in July 2022. This meeting was conducted in person at NHRC and lasted about four hours.

We revised the item list based on the expert reviews. Then, we conducted **cognitive interviews** with potential respondents to assess their understanding of the questions, evaluate problems with the wording of questions, and determine whether they were relevant and context-appropriate[59]. Trained research assistants recruited participants who had recently received ANC (pregnant or within 6 months of giving birth) from health facilities and conducted one-on-one interviews with them using the revised questions. Interviews were conducted at a convenient time and location for the participants in English and two local languages (Gurune and Kasem) following a training that included appropriate translations for the questions. Participants were told that their help was needed to develop the tool and that they should be comfortable recommending changes to how the questions were asked. They were then asked to respond to each question, followed by probes to understand why they answered the way they did, concerns with the wording of the questions, the relevance of the questions to their care experience, and any suggestions they had for improving the questions. We used probes instead of “thinking out loud” because these have worked better in previous cognitive interviews in similar settings. Interviews were recorded, and research assistants were engaged to debrief and make needed changes after six interviews (two in each language). Six additional interviews were conducted, during which only minor changes were suggested. Twelve cognitive interviews were conducted in total in October 2022, meeting the recommended sample size of at least 10 for cognitive interviews[60, 61].

Following revisions from the cognitive interviews, the scale items were integrated with a questionnaire including demographic and other questions, and the full questionnaire was **pretested** to identify any final issues with the scale items and the full questionnaire. We first pretested the revised tool with 12 pregnant and postpartum women, made relevant changes, and then pretested with another 12 women, which meets the recommended sample size of 15 to 30 for pretesting[62]. No significant changes were identified during the second set of pretests.

### Survey

The final questionnaire was administered in a cross-sectional survey to obtain quantitative data to assess the psychometric adequacy of the scale. Eligible participants were women in the third trimester of pregnancy or who had given birth within the last six months and who received ANC during their pregnancy. The surveys were conducted with 600 women (300 prenatal and 300 postpartum) in August and September 2023. Sample size estimation was based on the rule of thumb of about 5–10 subjects per item on the scale, with 300–500 considered good and 500 and above considered very good, regardless of the number of items[47, 63]. Following an analysis of data from this first survey, the questions were revised and administered as part of the baseline surveys in the Caring for Providers to Improve Patient Experience (CPIPE) trial [50] to 2000 postpartum women in Ghana and Kenya (1000 per country and ~ 500 per region or county) between March and October 2024. Eligible participants were postpartum women who had given birth within the 12 weeks preceding the survey in the 40 study facilities. For both surveys, trained research assistants recruited and interviewed women in study facilities and surrounding communities. Using the delivery registers from the study facilities, the research assistants identified eligible women and contacted them for interviews. Additionally, women who had just delivered and were still in the facility or those who were attending postnatal care (PNC) appointments were recruited. Participants were told about the study, and those who consented to participate were invited to participate in one-on-one interviews at a time and location that was convenient to them. We used a convenient sampling approach where all eligible women identified were interviewed until the required sample size was achieved. The surveys were programmed into REDCap, and data was entered using a tablet.

All research participants at each stage provided written informed consent and received a small token of appreciation (two cakes of soap in Ghana and Ksh 400 (~$3) in Kenya). Ethical approval was obtained from the Institutional Review Boards of the University of California, San Francisco (UCSF) and the Navrongo Health Research Center (NHRC) for the validation study and from UCSF, NHRC, and the Kenya Medical Research Institute for the CPIPE trial.

### Psychometric analysis

We assessed the scale’s construct and criterion validity and internal consistency reliability[47, 64]. We used inter-item correlations and factor analysis for item reduction and to assess construct validity—the degree to which the items represent the underlying conceptual structure. We first examined distributions of the items to note items with very low or high occurrences and to recode as needed. Negatively worded items were recoded to ensure higher numbers represent more person-centered care. Further, as in prior analyses, to ensure that all response options ranged from 0 to 3 for scoring, we took the conservative approach that assumes a positive, but not perfect, for not applicable responses. All items that had a “not applicable” or another fourth response were thus recoded to the upper middle category (2: most of the time, for positively worded items and 1: a few times, for negatively worded items)[45]. Next, we examined the correlations between individual items to identify items with very low and high correlations and the average inter-item correlation, aiming for an ideal average inter-item correlation between 0.20 and 0.40[65].

We then conducted iterative exploratory factor analysis (EFA) with the initial Ghana sample, using principal factoring with oblique rotations, which allow for correlation between the rotated factors, given that person-centered care domains are theoretically related[66]. We used the Kaiser-Meyer-Olkin (KMO) measure of sampling adequacy to assess the suitability of the variables for factor analysis, aiming for values between 0.8 and 1; Kaiser’s rule of retaining only factors with eigenvalues exceeding one and the “break” in the scree plot to determine the number of factors; and factor loadings and uniqueness to assess the performance of individual items, using a cut-off of < 0.3 for low loadings and > 9.0 for high uniqueness to mark items for potential removal (unless there was a strong conceptual rationale for their inclusion)[48, 66, 67]. Internal consistency reliability was assessed using Cronbach’s alpha, aiming for values ≥ 0.7[47, 64].

Following exploratory factor analysis, we conducted confirmatory factor analysis (CFA) using the CPIPE baseline surveys in Kenya and Ghana to assess if the three-factor structure could be uniformly applied across settings. In model building, we allowed error terms to correlate between items within the same proposed domain and used modification indices to systematically include the covariances of the errors. We assessed the goodness-of-fit of the full and reduced scales and subscales by estimating the root mean square error of approximation (RMSEA), p of close fit (pclose), comparative fit index (CFI), Tucker-Lewis index (TLI), and the Standardized root mean squared residual (SRMR) [68–70].

The responses for the final set of items are added to create summative scores, with scores standardized by dividing the mean score by the maximum possible scores (e.g., for a 36-item scale, the maximum score is 108 [36*3] and multiplying by 100). This creates a standardized score ranging from 0–100, where 0 is the worst and 100 is the best. Since there is no gold standard measure of PCANC, we assessed criterion validity—whether the measure is related to other measures in theoretically predictable ways—by examining how scores on the scale are associated with satisfaction and perceived quality of ANC and intent to use the same ANC facility in the future using cross-tabulations and linear regression. We assessed the criterion validity of the short scale by examining its correlation with the full scale and these measures.

## Results

### Conceptual adequacy

During the Initial review of the items by the study team, we retained all 34 items from the PCPC-US scale on assessment of face validity—at face value, they all appeared relevant to the experiences of women everywhere. We also added nine items we identified as relevant to ANC in LMICs from the literature, bringing the total number of items to 46. These 46 items were then sent out for expert reviews. During the expert review meeting, all items were deemed relevant and recommended to be maintained except for three questions. For the question, “Did providers knock on your room’s door and wait for a response before entering,” It was agreed by all that it was not relevant because in the setting, the provider is usually seated in the consulting room, and the patient walks in. This question was thus excluded at this stage. Several people also suggested removing the items on “*preferred clinic” and “preferred provider”* because they were generally not feasible in the setting and thus not considered highly relevant. However, a few recommended that they should be maintained. These two items were, therefore, marked for potential deletion later depending on their performance in subsequent phases. All respondents rated the tool as comprehensive or very comprehensive, which, together with the individual item ratings, indicated high content validity. There were, however, various suggestions to reword some questions and recommendations to include questions on physical and financial accessibility of services and counseling on specific issues. This increased the number of items to over 60, although some questions recommended for inclusion were not framed in a way that could facilitate scoring as part of a scale (e.g., questions on how much it costs to pay for services).

During cognitive interviews, most participants rated all items as important, with recommendations made to improve the wording of some items and to reorder some questions for better flow in the first round of interviews. No substantive changes were recommended during the second set of cognitive interviews. At the end of cognitive interviews, there were 68 questions, including 47 questions related to the three conceptual domains from the PCPC-US scale: four questions on wait time for various services, six questions on communication regarding specific ANC services, seven questions on counseling on specific issues, and four questions on perceived physical and financial accessibility. These items were all included in the questionnaire for pretesting and administered in the survey.

### Psychometric adequacy

#### Initial validation sample

The analytic sample for the initial psychometric analysis using data from only Ghana was 595 because of incomplete data from five respondents. The demographics of respondents in the analytic sample are shown in Table 1. The average age was 27 years, with 83% between 20 and 34 years old. Most respondents were married or partnered (93%) and had one to three children (73%). Most had less than a college education (86%) and earned or received less than 300 GHS (<$30 at the time) (76%) a month. Distributions of other variables are shown in Table 1.

Distributions for individual PCANC items for the initial sample are shown in Appendix 1. In general, most questions had responses across the four response options. However, most of the negatively worded items (neglect, verbal abuse, discrimination, physical abuse, bribe) had a very low frequency of occurrence, with 94% or more responding “no never” to a negative occurrence. A few questions were also not applicable (NA) to all respondents, with > 30% NA for wait time to retrieve folder and family treated with respect and >15% NA on wait time for drugs and labs, support when needed help, support to deal with emotional wellbeing, and preferred ANC provider and clinic. The correlations between the full set of items varied widely from negative to 0.8. Of note was that the wait time and negatively worded variables negatively correlated with most other items.

The KMO values ranged from 0.6 to 0.9, with an overall KMO of 85, indicating suitability for factor analysis. Initial exploratory factor analysis with all 68 items yielded nine factors with eigenvalues of >1, accounting for 81% of the cumulative variance, although the scree plot suggested 3–4 factors. Most items from the original PCPC-US scale loaded on the first two factors, and the items on financial and physical accessibility and counseling and communication on specific tests and procedures loaded on the other factors. Therefore, most of these items were removed at this point as the analysis confirmed they were conceptually different from the rest of the items. The six questions about specific examinations and tests were recoded to combine into two variables after being told the results. All the perceived wait time questions also loaded poorly with the rest of the items. However, because of the importance of timeliness to responsiveness, we decided to retain the item on wait time to see the clinical provider and wait time for getting labs done and medications from the pharmacy (because the team thought these were important sources of delay in the facility). We dropped the question on wait time for folders (following clarifications that while some facilities keep folders, in many cases, the antenatal record that mothers keep is used; hence, mothers do not wait for their folders, explaining the high proportion of NA on that question). The two variables on wait time for labs and drugs were, however, recoded to one item on wait time to get labs or drugs because of the large proportion of NA responses for each. We also decided to combine the questions on whether they were asked about their emotional and mental wellbeing because they were interpreted similarly, had similar distributions, and were strongly correlated (r=0.76). Other items from the initial PCPC-US scale loaded poorly were retained at this stage, yielding 50 items for subsequent analysis.

EFA of the 50 items yielded five factors with eigenvalues greater than 1, accounting for 78% of the cumulative variance. However, most items were loaded on three factors, and the scree plot supported a single dominant factor. Most items had a loading of >0.3 on one of the three factors and on the single factor when constrained to this factor structure (appendix 2). The negatively worded items (discrimination, physical abuse, bribe, and neglect), which had low frequencies in the sample, as noted above, continued to load poorly (<2) and had high uniqueness (>0.95), so they were dropped at this stage. In iterative EFAs, more items were dropped based on different rationales to produce a shorter scale. This included removing items based on expert reviews because of low relevance (preferred clinic and provider) and those that had loadings of <0.3 and uniqueness >0.90 (pressured). Further, for items that were strongly correlated or captured a similar concept, only one was retained, with the one that was conceptually broader or had more favorable loading and uniqueness maintained (e.g., for “explained medicines” and “understood medicines,” we kept “explained medicines;” for “explained examinations and procedures” and “understood examinations and procedures”; we kept “explained examinations and procedures”; for asked “if they had questions “and “encouraged to ask questions,” we kept “encouraged to ask questions”; for “could ask any questions” and “held back from asking questions,” we kept “could ask any question”; for “language they could understand” and “language level they could understand,” we kept “language level they could understand”; and for “showed they cared” and “best care” we kept “best care”). “Family respected” was also removed because of the high NA responses (47%), as most respondents did not have a family with them. Some items with moderate loading (<3 but >2: privacy, verbal abuse, cleanliness, enough staff, time with the provider, asking about their birth plan, asked physical wellbeing) were, however, retained in this phase to assess potential removal in subsequent phases. The wait time items were also retained despite poor loading because of the conceptual relevance of timeliness. This yielded 36 items.

**Figure 1:**
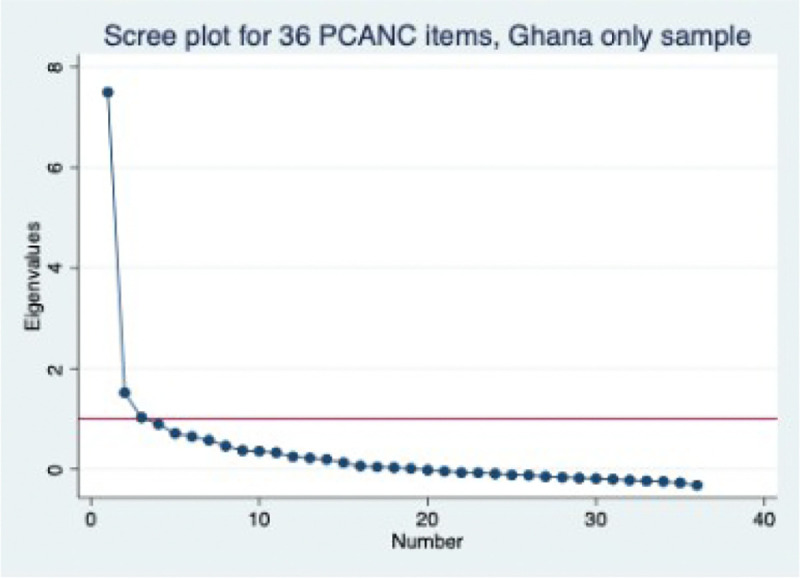


Factor analysis of the 36 items (Table 2) yields factors with eigenvalues of >1, accounting for 81% of the cumulative variance, but with one dominant factor (Figure 1) with an eigenvalue of 7.5, accounting for 61% of the cumulative variance. All items have loadings ≥3 on the three factors except for the two wait time variables with loadings less than two and six items with loadings between 2 and 3 (privacy, verbal abuse, language level, time with provider, cleanliness, and enough staff). The uniqueness for most items was <0.9 except for five of these items, with a uniqueness of 0.95 for the wait time variables on the three-factor structure (Table 3). When the analysis was restricted to the single factor structure, all items had loadings of >0.3 except 10 items (the two wait time variables have loadings less than two; and eight items—privacy, verbal abuse, time with provider, cleanliness, enough, birth plan, told exams, told results—have loadings between 2 and 3). The best fit in this sample was thus 28 items for a multidimensional scale and 26 for a unidimensional scale. Sequential exclusion of all 10 items with low loading in the 3 or single-factor structure did not significantly change the Cronbach’s alpha, and all versions had correlations >0.96. Thus, we proceeded with the 36 items for testing in subsequent samples.

As in prior validation studies, because the loading of the items does not group into clean conceptual categories, we created sub-scales based on conceptual groupings adapted from the World Health Organization’s experience of care domains [71]. This gives three subscales for “dignity and respect,” “communication and autonomy,” and “responsive and supportive care.” Factor analysis of items in each subscale yielded a single factor, with all items loading at >0.4 on the single factor and with uniqueness <0.8, except for 10 items noted above (Table 3).

The full 36-item scale has Cronbach’s alpha of 0.89, and all subscales have Cronbach’s alphas >0.7 (Table 3). This does not change with the exclusion of the wait time and time with provider items. The average interitem correlation is 0.18 for the full 36-item scale and 0.27, 0.20, and 0.15 for the “dignity and respect,” “communication and autonomy,” and “responsive and supportive care” sub-scales, respectively. These increased to 0.20 and 0.22 for the full scale and “responsive and supportive care” subscale, respectively, when the wait time and time with provider variables are excluded (i.e., 33-item scale).

The average standardized score for the 36-item PCANC scale is 77.6 out of 100, with scores of 86.4, 73.5, and 76.8. respectively for the “dignity and respect,” “communication and autonomy,” and “responsive and supportive care” sub-scales for the Ghana test sample (Table 4). Cross-tabulating and regressing the full scale on patients’ ratings of satisfaction with ANC services, general perceived quality of ANC, and whether they would receive ANC in the same facility if they were to be pregnant again showed increasing PCANC scores with higher ratings of satisfaction, perceived quality of care, and intent to receive ANC in the same facility (Table 4)

#### Confirmation sample:

Among the CPIPE baseline participants, 1,992 received ANC, with incomplete PCANC data from four additional respondents, which yielded an analytic sample of 1,988 for the confirmation sample. The demographics for the validation sample are shown in Table 5 by country. The average age was 26.9 (SD=5.8) and 25.4 (SD=5.6) years for Ghana and Kenya respectively. Most respondents were married or partnered (91.5% and 79.8% for Ghana and Kenya, respectively) and had 1 to 2 children (55.4% and 51.7% for Ghana and Kenya, respectively). Only about 10% of respondents had a college education (9.5 and 11% for Ghana and Kenya, respectively), and most earned or received <$100 a month (1000 or less GHS for 61.3% in Ghana and 10000 or less KES for 68.7% in Kenya).

Forty-six PCANC questions were included in the CPIPE trial baseline surveys, including the 36 items from the psychometric analysis, nine other items that the team thought were important to reassess in a new sample, and a new question on the accessibility of washrooms because some respondents noted there was no washroom in the facility in response to the question on cleanliness in the previous survey. The distribution of these items in this sample is shown in Appendix 3. An initial exploratory factor analysis with this sample confirmed that the nine items included that performed poorly in the initial Ghana sample continued to perform poorly in the validation sample (appendix 4), so these were finally dropped. In addition, the item on time with a provider continued to perform poorly in this sample, with a uniqueness of 1 on the single-factor structure. So, this was also dropped at this point. The two wait time questions, however, loaded adequately with the three-factor structure, although they were the only two items loading on the third factor and loaded poorly in the single-factor structure. The new question on the accessibility of washrooms also performed well. A decision was thus made to retain these three items, which maintained a 36-item scale.

**Figure 2:**
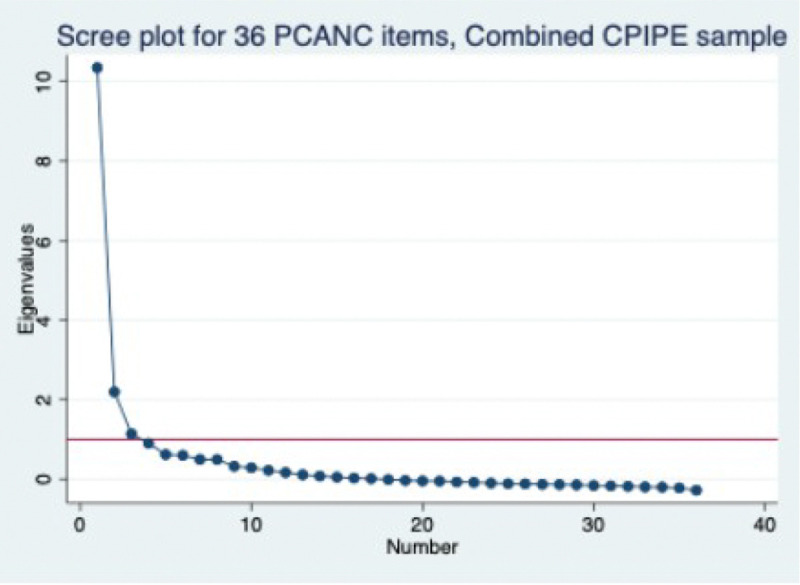


The average KMO for the 36 items is 0.94 (range of 0.56 to 0.97), with three factors >1, which accounts for 87% of the cumulative variance, and one dominant factor (Figure 2), accounting for 66% of the cumulative variance. All 36 remaining items were loaded at >0.3 on the three factors except for verbal abuse and auditory privacy, which had loadings between 2 and 3 (appendix 6) but were maintained because of their strong conceptual relevance. As in the test sample studies, we created the sub-scales based on conceptual groupings for “dignity and respect,” “communication and autonomy,” and “responsive and supportive care.” Factor analysis of items in each subscale yielded a single factor, with all items loading at >0.4 on the single factor and with uniqueness <0.8, except for verbal abuse, language, and the time variables (appendix 5). Given the very poor loading of the two wait time variables, we also tested a 34-item scale that excludes those two variables. Further, to provide a smaller subset of items for instances where all 36 items might not be feasible to administer, we used an iterative approach where all items with factor loading <.3 were dropped, and additional items dropped based on our assessment of the strength of their relevance and similarity to other retained items, leading to a 20-item short scale (items shown in table 6).

These 36-item and 20-item scales with the three sub-scales were then evaluated in the Confirmatory Factor Analysis (CFA) for the combined sample and each country. The standardized factor loadings from the CFA for the observed items mainly ranged from 0.08 to 0.76, with only one item (wait time for labs or drugs) having a negative factor loading. All factor loadings significantly differed from 0, suggesting that most observed items positively correlated with the latent variables (Table 6). The model in Ghana alone had factor loadings that ranged from −0.84 to 0.84, and in Kenya, the factor loadings ranged from 0.08 to 0.71, with all the factor loadings significantly different from 0. The Goodness-of-Fit Indicators of the three-factor confirmatory model indicated the best-fitted model was the model with covariances of the error terms of 10 or more for the two countries combined indicated by RMSEA<0.08, p-close >0.05, CFI and TLI>.9, and SRMR <0.05 (Table 7 and Appendix 6).

The full 36-item scale has Cronbach’s alpha of 0.92, increasing to 0.93 when the two wait time variables are dropped, and that of the 20-item scale is 0.89. All subscales have Cronbach’s alphas >0.75 (Table 8). The average standardized score for the 36-item PCANC scale for the validation sample is 71.5 out of 100, with a similar score of 71.1 for the 34-item version and 70.6 for the 20-item version. The scores for the “dignity and respect,” “communication and autonomy,” and “responsive and supportive care” sub-scales are 81.2, 65.3, and 72.8, respectively, for the full sample, and that by country is shown in Table 8.

Cross-tabulating and regressing the full scale on patients’ ratings of satisfaction with ANC services, general perceived quality of ANC, and whether they would receive ANC in the same facility if they were to be pregnant again showed increasing PCANC scores with higher ratings of satisfaction, perceived quality of care, and intent to receive ANC in the same facility (Table 9). Scores on the 36, 34, and 20-item scales are strongly correlated (r=1.0 between the 34 and 36-item versions and 0.97 and 0.98 with the 20-item scale), suggesting good criterion validity for the shorter versions.

Table 10 shows all the PCANC items and the reasons for their inclusion/exclusion in the scale’s final long and short forms.

## Discussion

We developed a PCANC scale with good content, construct, criterion, and known-group validity that is relevant to LMICs using a rigorous scale development process. The initial phases of scale development, including the literature reviews, expert reviews, and cognitive interviews, ensured content validity, the most important form of validity for any measurement tool [56, 57]. The psychometric analysis using a sample of pregnant and postpartum women in Ghana and Kenya yielded a 36-item comprehensive scale with adequate structural validity, three subscales for dignity and respect, communication and autonomy, and responsive and supportive care. We also developed a shorter 20-item version for instances where the 36 items might be too much. The full scales and subscales have good internal consistency reliability, with Cronbach’s alpha > 0.8 for the full scale and > 0.7 for the subscales. Higher scores on the scale are associated with higher satisfaction, perceived quality of care, and intent to use the facility in the future, as hypothesized, indicating criterion validity.

In general, the PCANC scale developed for LMICs performed similarly to the original PCPC-US scale[45]. The PCPC-US scale had 34 items on the full scale compared to 36 items on this scale. Most (26) items in the PCPC-US scale were retained in this scale, with nine items (knocking on the door, information showing they cared, family respected, physical abuse, discrimination, hold back on asking questions, coerced, preferred clinic, and provider) excluded. These are replaced with 11 items in the current version (time to get labs and drugs, auditory privacy, attention when needed help, greeted, friendly care, received best care, communication on tests and procedures, assessment of physical wellbeing, competence, availability of washrooms, cleanliness, and enough staff) not included in the PCPC-US scale. These additional items represent issues that are relevant in LMICs. The same three sub-scales are used in both versions as they represent relevant constructs globally.

Although wait time, considered very relevant during expert reviews, performed poorly in the psychometric analyses, it is maintained in the current version of the scale. Timeliness is an important aspect of person-centered care, particularly related to responsiveness, and should be measured in any work to improve person-centered care. Including timeliness thus increases the content validity of the scale. Timeliness can, however, be considered a separate construct reflected in the loading of the two wait time variables. This is also reflected in their being presented as separate domains of healthcare quality (Safe, Effective, Patient-centered, Timely, Efficient, and Equitable)[8]. Thus, we could have a 34-item scale with the two items on timeliness. In addition to safety, we have maintained them in one scale because they are unlikely to be measured if excluded or if each domain has a different scale. The current scale thus includes Safety, Patient-centeredness, and Timeliness, which, when used as an outcome, can be used to assess the effectiveness and efficiency of interventions and, when used with equity stratifies, can be used to assess equitable healthcare quality.

Many of the negatively worded items (physical abuse, neglect, discrimination, and neglect) were considered relevant; however, they did not perform even moderately well, likely because of their low frequency of occurrence in our sample and were thus excluded. These items may perform better when overt mistreatment during ANC is high. Thus, we recommend assessing all 46 relevant PCANC items in future validation studies to develop the best set of items that perform well across settings. Further, many questions excluded from the scale, including accessibility, service provision, counseling, and overt mistreatment, are still relevant to high-quality PCANC. Thus, although not included in scoring in the PCANC scale, these can be examined separately to get a full picture of the quality of ANC.

Like prior scales, the PCANC scale includes a mix of subjective (e.g., felt treated with respect) as well as more objective questions (e.g., procedures explained)[26, 45]. Both types of questions are important in assessing person-centered care in a way that accounts for people’s subjective experiences and reflects what happens during the encounter independent of people’s expectations[72]. The PCANC scale is, therefore, an actionable patient-reported experience measure that can inform quality improvement activities at various levels of the health system. The full scale captures all the key domains of PCANC and can thus be used for comprehensive assessments. The sub-scales can be used independently where some domains are deemed more relevant. The response format ensures that people’s responses are captured on a continuum, increasing the tool’s responsiveness to detect change. The PCANC scores can thus be tracked to examine intervention effects or to assess change over time and across settings.

## Strengths and Limitations

The PCANC scale is based on a strong theoretical and empirical foundation, given that it is built on prior work, including the PCMC and PCPC-US scales. The rigorous adaptation process based on standard scale development steps ensured that the final scale had high content validity and was relevant to the target population. A potential limitation is that it may not capture issues of relevance to other LMICs, given that the initial adaptation process was only in Ghana and the final validation samples are only from Ghana and Kenya. However, based on our literature review, we believe the items will apply in most other LMIC settings as they were directly applicable in Kenya without a need for additional adaptation. What might be missing are items that are unique to different settings. For example, the overt mistreatment items fell off because of the low frequency of occurrence of these behaviors in the study samples. They will, however, be important where overt mistreatment during ANC exists. Future testing in diverse settings is therefore needed. The most important but challenging part of tool development is ensuring content validity. Yet most validation efforts focus on just psychometric adequacy. The pool of items we developed will thus provide a starting point for future psychometric assessments in different settings. Given the rapid uptake of the PCMC scale and validation in other settings after the initial validation, we believe this validation study in two LMIC countries will spur further validation in other LMIC settings. Other drawbacks include the length of the scale and associated participant burden. We address this by proposing a shorter version.

## Conclusions

We present a valid and reliable comprehensive tool to measure the key domains of PCANC in LMIC contexts. Like prior person-centered tools, this tool will be valuable for needs assessment to identify gaps in PCANC to inform interventions and for evaluation to assess the effect of interventions on PCANC. In addition, it can be used for research to examine predictors to identify inequities in PCANC and assess its impact on maternal and neonatal outcomes to improve advocacy. It could also be used for routine monitoring for quality improvement and accountability. PCANC is critical to achieving the vision for maternal and newborn health. However, monitoring progress toward this vision will be difficult without measurement tools. This study thus addresses a critical gap in efforts to measure and provide high-quality ANC in LIMICs.

## Figures and Tables

**Table 1:** Characteristic of the initial validation study population, Ghana, N=595

Characteristic	Frequency (%)
Age in years: Mean (SD)	26.94 (5.57)
Below 20 yrs	35 (5.9)
20–24	208 (35.0)
25–29	164 (27.6)
30–34	122 (20.5)
35–39	55 (9.2)
40 or more	11 (1.8)
Marital status	
Married/Partnered	553 (92.9)
Single	34 (5.7)
Widowed/Divorced/separated	8 (1.3)
Parity	
None	75 (12.6)
One	195 (32.8)
Two	151 (25.4)
Three	90 (15.1)
Four	53 (8.9)
5 or more	31 (5.2)
Pregnant or give birth in the last 6 months	
Yes, currently pregnant	297 (49.9)
Yes, gave birth in the last 6 months	298 (50.1)
Months of pregnancy (Pregnant participants)	
6 months	43 (14.5)
7 months	99 (33.4)
8 months	99 (33.4)
9 months	53 (17.9)
10 months	2 (0.7)
Months of postpartum (Postpartum participants)	
3 wks or less	59 (19.8)
4–7 wks	61 (20.5)
8–11 wks	39 (13.1)
3 months	52 (17.4)
4 months	38 (12.8)
5 months	30 (10.1)
6 months	19 (6.4)
Highest grade completed at school	
None	29 (4.9)
Primary or less	128 (21.5)
Post-primary/vocational	196 (32.9)
Secondary	188 (31.6)
College/University	53 (8.9)
Refused to answer	1 (0.2)
Partner's highest grade completed	
None	47 (7.9)
Primary or less	111 (18.7)
Post-primary/vocational	140 (23.5)
Secondary	151 (25.4)
College/University	111 (18.7)
Refused to answer	35 (5.9)
Occupation	
Farming	104 (17.5)
Trading/selling	107 (18.0)
Hairdressing/dressmaking/Craftsmanship	142 (23.9)
Housewife/unemployed	144 (24.2)
Teacher/Student	51 (8.6)
Other	47 (7.9)
Partner's occupation	
Farming	189 (31.8)
Trading/selling	62 (10.4)
Hairdressing/dressmaking/Craftsmanship	78 (13.1)
unemployed	27 (4.5)
Teacher/Student	76 (12.8)
Motor/Driver/Mechanic	65 (10.9)
Mason/Electrician/Plumbing	30 (5.0)
Other	68 (11.4)
Read and write	
No, cannot read and write	147 (24.7)
Yes, but with some difficulty with reading or writing	210 (35.3)
Yes, can read and write very well	238 (40.0)
Money earned/received in a month (GHS)	
None/undisclosed	64 (10.8)
100 or less	200 (33.6)
101–200	111 (18.7)
201–300	77 (12.9)
301–400	35 (5.9)
401–500	45 (7.6)
More than 500	63 (10.6)
Household wealth quintile	
First (poorest)	187 (31.4)
Second (poorer)	56 (9.4)
Third (middle)	129 (21.7)
Fourth (richer)	117 (19.7)
Fifth (richest)	106 (17.8)
Household member work in a health facility	
No	517 (87.0)
Yes	77 (13.0)
Religion	
Christian	535 (89.9)
Muslim	40 (6.7)
Traditionalist	18 (3.0)
Other	2 (0.3)
Ethnicity	
Nankani/Frafra	291 (48.9)
Kasem	211 (35.5)
Builsa	63 (10.6)
Others	30 (5.0)
Timing of first ANC	
2 months or less	224 (37.6)
3–4	273 (45.9)
5–6	83 (13.9)
7 or more	15 (2.5)
Reason for first ANC visit	
Just for a routine checkup	475 (79.8)
Because of a problem	120 (20.2)
Frequency of ANC visits	
2 or less	32 (5.4)
3–5	184 (30.9)
6–8	207 (34.8)
9 or more times	172 (28.9)
Highest-level ANC facility	
Government Hospital	105 (17.6)
Government HC	195 (32.8)
Other lower-level facility	240 (40.3)
Private or mission facility	55 (9.2)
Highest level provider received ANC from	
Doctor or Medical assistant/Physician assistant/Clinical Officer	28 (4.7)
Midwife/nurse	567 (95.3)

**Table 2:** Exploratory factor analysis of 36 retained items, Ghana, N=595

	3 factor structure for full scale				single factor structure for full scale		Single factor by subscale	
*Subscale/items*	*Factor1*	*Factor2*	*Factor3*	*Uniqueness*	*Factor1*	*Uniqueness*	*Factor1*	*Uniqueness*
**Dignity and respect**								
Greeted	0.38		0.39	0.60	0.56	0.69	0.52	0.73
Treat you with respect	0.73			0.50	0.65	0.58	0.76	0.43
Friendly care	0.70			0.50	0.67	0.56	0.78	0.39
Knowledge valued	0.71			0.52	0.65	0.58	0.64	0.59
Privacy/not exposed			0.27	0.90	0.23	0.95	0.24	0.94
Info confidentiality			0.53	0.66	0.42	0.82	0.41	0.83
Auditory privacy	0.47			0.70	0.49	0.76	0.50	0.75
Verbal abuse	0.27			0.91	0.30	0.91	0.29	0.92
**Communication and Autonomy**								
Introductions by provider			0.35	0.72	0.47	0.78	0.48	0.77
Called preferred name			0.32	0.83	0.34	0.88	0.33	0.89
Heard and listened to	0.66			0.60	0.58	0.67	0.49	0.76
Involved in decisions			0.38	0.69	0.49	0.76	0.47	0.78
Consent	0.63			0.64	0.55	0.70	0.53	0.72
Explain exams/procedures	0.49			0.68	0.53	0.72	0.57	0.68
Explain medications	0.40			0.75	0.49	0.76	0.55	0.69
Told examination results		0.34	0.40	0.75	0.19	0.96	0.26	0.93
Told test results		0.35	0.39	0.75	0.16	0.97	0.22	0.95
Could ask any questions	0.42			0.76	0.46	0.79	0.46	0.78
Encouraged questions	0.34	0.45		0.59	0.55	0.69	0.61	0.63
Questions were answered	0.33	0.33		0.65	0.56	0.68	0.59	0.66
Check understood	0.34	0.30		0.60	0.61	0.63	0.64	0.60
Language level they understood			0.24	0.85	0.34	0.88	0.32	0.89
Asked about birth plan		0.32		0.88	0.24	0.94	0.27	0.93
**Responsive and Supportive care**								
Wait time for provider			0.11	0.98	0.10	0.99	0.12	0.99
Wait time for labs and drugs			0.12	0.95	−0.12	0.99	−0.14	0.98
Time with provider	0.28			0.92	0.20	0.96	0.17	0.97
Received best care	0.58			0.58	0.63	0.61	0.63	0.60
Physical wellbeing assessed	0.30			0.75	0.47	0.77	0.51	0.74
Mental/emotions wellbeing assessed		0.67		0.57	0.38	0.85	0.46	0.79
Resources for emotional/mental wellbeing		0.60		0.61	0.42	0.83	0.54	0.71
Attention when needed help		0.31		0.79	0.42	0.82	0.44	0.80
Trust	0.50			0.68	0.56	0.69	0.58	0.67
Competence	0.57			0.56	0.65	0.58	0.63	0.60
Cleanliness			0.25	0.87	0.19	0.96	0.22	0.95
Safe	0.40			0.77	0.38	0.86	0.40	0.84
Enough staff		0.28		0.88	0.22	0.95	0.29	0.92

**Table 3:** Characteristics of scale items, Ghana, N=595

		*Internal Consistency*		*Standardized scores*			
	Number of items	Average interitem correlation	Cronbach's alpha	Mean	Std. dev.	Min	Max
Full 36-item PCANC scale	36	0.18	0.89	77.57	11.87	22.22	96.30
Dignity and Respect subscale	8	0.27	0.74	86.44	14.99	16.67	100.00
Communication and autonomy subscale	15	0.20	0.79	73.49	14.96	15.56	100.00
Responsive and supportive care subscale	13	0.15	0.70	76.82	11.59	28.21	100.00

**Table 4:** Crosstabulation and Linear Regression on PCANC score, Ghana, N=594

	*Crosstab*			*Linear Regression on PCANC score*			
	N	Mean	SD	Coefficient	p-value	[95% conf. interval]	
**Satisfaction with ANC**							
Dissatisfied	5	54.44	21.4	−19.28	0.03	−36.17	−2.38
Neither satisfied nor dissatisfied	19	57.26	13.1	−16.46	0.00	−22.36	−10.57
Satisfied	284	73.72	11	ref			
Very satisfied	287	83.12	8.54	9.40	0.00	7.78	11.03

**Perceived quality of ANC**							
Very Poor	2	43.06	29.5	−27.34	0.07	−56.46	1.79
Poor	9	54.12	13.4	−16.28	0.00	−24.73	−7.82
Fair	20	59.49	13.4	−10.90	0.00	−16.85	−4.95
Good	183	70.39	10.5	ref			
Very good	236	80.61	8.34	10.22	0.00	8.36	12.09
Excellent	143	86.23	5.93	15.84	0.00	14.03	17.66

**Will return for ANC in future**							
No, never	63	70.56	15.3	−9.07	0.00	−12.96	−5.18
Yes, somewhat	56	67.92	12.5	−11.71	0.00	−15.09	−8.33
Yes, definitely	475	79.63	10.3	ref			

**Table 5:** Demographics for CPIPE trial Baseline for Ghana and Kenya, N=1988

Characteristic	Overall (N=1988)	Country		
		Kenya (N=996)	Ghana (N=992)	p
Age in years: Mean (SD)	26.20 (5.78)	25.45 (5.65)	26.96 (5.80)	<0.001
Below 20 years	216 (10.9)	133 (13.4)	83 (8.4)	<0.001
20–24	683 (34.4)	384 (38.6)	299 (30.1)	
25–29	538 (27.1)	243 (24.4)	295 (29.7)	
30–34	338 (17.0)	148 (14.9)	190 (19.2)	
35–39	165 (8.3)	72 (7.2)	93 (9.4)	
40 or more	38 (1.9)	12 (1.2)	26 (2.6)	
Refused to answer	10 (0.5)	4 (0.4)	6 (0.6)	
Marital status									
Single	259 (13.0)	177 (17.8)	82 (8.3)	<0.001
Married/Partnered	1,702 (85.6)	795 (79.8)	907 (91.4)	
Widowed/Divorced/separated	25 (1.3)	23 (2.3)	2 (0.2)	
Refused to answer	2 (0.1)	1 (0.1)	1 (0.1)	
Parity									
1–2	1,063 (53.5)	515 (51.7)	548 (55.2)	0.006
3–4	652 (32.8)	322 (32.3)	330 (33.3)	
5–6	214 (10.8)	118 (11.8)	96 (9.7)	
7 or more	59 (3.0)	41 (4.1)	18 (1.8)	
Months of postpartum									
3 wks or less	875 (44.0)	410 (41.2)	465 (46.9)	<0.001
4–7 wks	560 (28.2)	310 (31.2)	250 (25.2)	
8–11 wks	447 (22.5)	236 (23.7)	211 (21.3)	
3 months	105 (5.3)	39 (3.9)	66 (6.7)	
Highest grade completed at school									
None	166 (8.4)	3 (0.3)	163 (16.4)	<0.001
Primary or less	643 (32.3)	476 (47.8)	167 (16.8)	
Post-primary/vocational	255 (12.8)	53 (5.3)	202 (20.4)	
Secondary	719 (36.2)	354 (35.5)	365 (36.8)	
College/University	205 (10.3)	110 (11.0)	95 (9.6)	
Partner's highest grade completed									
None	138 (6.9)	3 (0.3)	135 (13.6)	<0.001
Primary or less	504 (25.4)	301 (30.2)	203 (20.5)	
Post-primary/vocational	195 (9.8)	103 (10.3)	92 (9.3)	
Secondary	486 (24.4)	212 (21.3)	274 (27.6)	
College/University	330 (16.6)	142 (14.3)	188 (19.0)	
Refused to answer	285 (14.3)	199 (20.0)	86 (8.7)	
Don’t Know	50 (2.5)	36 (3.6)	14 (1.4)	
Occupation				
Farming	256 (12.9)	127 (12.8)	129 (13.0)	<0.001
Trading/selling	352 (17.7)	190 (19.1)	162 (16.3)	
Hairdressing/dressmaking/Craftsmanship	233 (11.7)	50 (5.0)	183 (18.4)	
Housewife/unemployed	725 (36.5)	464 (46.6)	261 (26.3)	
Teacher/Student	241 (12.1)	128 (12.9)	113 (11.4)	
Others	181 (9.1)	37 (3.7)	144 (14.5)	
Partner's occupation				
Farming	538 (27.1)	194 (19.5)	344 (34.7)	<0.001
Trading/selling	249 (12.5)	156 (15.7)	93 (9.4)	
Hairdressing/dressmaking/Craftsmanship	229 (11.5)	145 (14.6)	84 (8.5)	
unemployed	108 (5.4)	51 (5.1)	57 (5.7)	
Teacher/Student	178 (9.0)	64 (6.4)	114 (11.5)	
Motor/Driver/Mechanic	335 (16.9)	229 (23.0)	106 (10.7)	
Mason/Electrician/Plumbing	10 (0.5)	4 (0.4)	6 (0.6)	
Others	341 (17.2)	153 (15.4)	188 (19.0)	
Read and write				
No, cannot read and write	322 (16.2)	16 (1.6)	306 (30.8)	<0.001
Yes, but with some difficulty with reading or writing	310 (15.6)	128 (12.9)	182 (18.3)	
Yes, can read and write very well	1,350 (67.9)	849 (85.2)	501 (50.5)	
Refused to answer	6 (0.3)	3 (0.3)	3 (0.3)	
Money earned/received in a month (Ghana)				
< =1000 cedis		−	605 (61.2)	.
>1000 to 5000 cedis		−	153 (15.5)	
>5000 to 10000 cedis		−	6 (0.6)	
Refused to answer		−	224 (22.7)	
Money earned/received in a month (Kenya)				
< =10000 KSH		684 (68.7)	−	.
>10000 to 50000 KSH		229 (23.0)	−	
>50000 to 100000 KSH		12 (1.2)	−	
>100000 to 150000 KSH		2 (0.2)	−	
>200000 KSH		1 (0.1)	−	
Refused to answer		67 (6.7)	−	
Household wealth quintile				
First	405 (20.4)	153 (15.4)	252 (25.4)	<0.001
Second	510 (25.7)	273 (27.4)	237 (23.9)	
Third	307 (15.4)	175 (17.6)	132 (13.3)	
Fourth	438 (22.0)	216 (21.7)	222 (22.4)	
Fifth	328 (16.5)	179 (18.0)	149 (15.0)	
Religion				
Christian	1,698 (85.4)	980 (98.4)	718 (72.4)	<0.001
Muslim	264 (13.3)	11 (1.1)	253 (25.5)	
Traditionalist	24 (1.2)	5 (0.5)	19 (1.9)	
Other	2 (0.1)	0 (0.0)	2 (0.2)	
Ethnicity in Kenya				
Luyya		30 (3.0)	−	.
Kuria		95 (9.5)	−	
Abusuba		5 (0.5)	−	
Kisii		29 (2.9)	−	
Luo		815 (81.8)	−	
Other		22 (2.2)	−	
Ethnicity in Ghana				
Kasem		−	69 (7.0)	.
Nankani/Frafra		−	309 (31.1)	
Builsa		−	149 (15.0)	
Nabdam		−	4 (0.4)	
Kusal		−	82 (8.3)	
Talensi		−	50 (5.0)	
Mampruli		−	150 (15.1)	
Other		−	178 (17.9)	
Refused to answer		−	1 (0.1)	
Timing of first ANC				
2 months or less	526 (26.5)	116 (11.7)	410 (41.3)	<0.001
3–4	854 (43.0)	432 (43.4)	422 (42.5)	
5–6	519 (26.1)	394 (39.6)	125 (12.6)	
7 or more	88 (4.4)	53 (5.3)	35 (3.5)	
Reason for first ANC visit				
Because of a problem	301 (15.1)	187 (18.8)	114 (11.5)	<0.001
Just for a routine checkup	1,681 (84.6)	803 (80.7)	878 (88.5)	
Don’t Know	4 (0.2)	4 (0.4)	0 (0.0)	
Not applicable	1 (0.1)	1 (0.1)	0 (0.0)	
Number of ANC visits				
2 or less	91 (4.6)	72 (7.2)	19 (1.9)	<0.001
3–5	859 (43.2)	683 (68.6)	176 (17.7)	
6–8	637 (32.0)	226 (22.7)	411 (41.4)	
9 or more times	387 (19.5)	10 (1.0)	377 (38.0)	
Don’t Know/missing	14 (0.7)	5 (0.5)	9 (0.9)	
Highest-level ANC facility				
Gov’t hospital	851 (43.0)	572 (57.5)	279 (28.4)	<0.001
Gov’t Health Center/other lower level	961 (48.6)	398 (40.0)	563 (57.3)	
Private or mission facility	165 (8.3)	24 (2.4)	141 (14.3)	
Highest level provider received ANC from				
Doctor/Clinical Officer	343 (17.3)	256 (25.7)	87 (8.8)	<0.001
Nurse/Midwife	1,639 (82.6)	738 (74.2)	901 (91.0)	
Non-skilled attendant/2DK	3 (0.2)	1 (0.1)	2 (0.2)	

**Table 6:** Unstandardized and Standardized Loadings for Three-Factor Confirmatory Model of the PCANC scale, N=1988

Latent variable / observed variable	Using 36-observed variables		Using 20-observed variables	
	Unstandardized (Standard error)	Standardized (Standard error)	Unstandardized (Standard error)	Standardized (Standard error)
**Dignity and respect**				
Greeted	1	0.62 (0.02)	1	0.64 (0.02)
Treat you with respect	0.77 (0.03)	0.69 (0.02)	0.77 (0.03)	0.72 (0.02)
Friendly care	0.82 (0.03)	0.70 (0.02)	0.82 (0.03)	0.72 (0.02)
Knowledge valued	1.09 (0.05)	0.75 (0.02)	-	-
Privacy not exposed	0.91 (0.05)	0.53 (0.02)	0.87 (0.05)	0.53 (0.02)
Info confidentiality	0.59 (0.04)	0.47 (0.02)	0.58 (0.03)	0.48 (0.02)
Auditory privacy	0.85 (0.05)	0.45 (0.02)	-	-
Verbal abuse	0.16(0.02)	0.23 (0.02)	-	-
**Communication and autonomy**				
Introductions by provider	1	0.34 (0.02)	1	0.34 (0.02)
Called preferred name	0.99 (0.10)	0.34 (0.02)	1.00 (0.10)	0.35 (0.02)
Heard and listened to	1.57 (0.12)	0.62 (0.02)	1.59 (0.12)	0.63 (0.02)
Consent	1.66(0.12)	0.53 (0.02)	1.68 (0.12)	0.54 (0.02)
Involved in decisions	2.03 (0.15)	0.68 (0.01)	2.10(0.15)	0.71 (0.02)
Explain exams/procedures	2.24 (0.16)	0.69 (0.02)	1.96(0.15)	0.62 (0.02)
Explain medications	2.14(0.15)	0.71 (0.01)	2.00 (0.14)	0.67 (0.02)
Told examination results	1.86(0.14)	0.57 (0.02)	-	-
Told test results	1.94 (0.15)	0.60 (0.02)	-	-
Could ask any questions	1.93 (0.14)	0.68 (0.01)	-	-
Encouraged questions	2.36(0.17)	0.72 (0.01)	2.17(0.16)	0.66 (0.02)
Questions were answered	1.78 (0.13)	0.68 (0.02)	-	-
Check understood information	2.27 (0.16)	0.72 (0.01)	2.23 (0.16)	0.71 (0.01)
language they understood	0.35 (0.04)	0.25 (0.02)	-	-
Asked about birth plan	1.37 (0.11)	0.40 (0.02)	-	-
**Responsive and supportive care**				
Received best care	1	0.76 (0.01)	1	0.65 (0.02)
Wait time for provider	0.12(0.03)	0.08 (0.02)	-	-
Wait time for labs or drugs	−0.18 (0.04)	−0.10(0.02)	-	-
Physical wellbeing assessed	0.82 (0.05)	0.43 (0.02)	-	-
Mental/emotions wellbeing assessed	1.28 (0.06)	0.55 (0.02)	1.21 (0.07)	0.44 (0.02
Resources for emotional/mental wellbeing	1.02 (0.06)	0.45 (0.02)	-	-
Attention when needed help	0.98 (0.04)	0.69 (0.01)	-	-
Trust	1.01 (0.03)	0.71 (0.01)	0.98 (0.03)	0.59 (0.02)
Competence	0.73 (0.03)	0.57 (0.02)	-	-
Washrooms	0.58 (0.05)	0.31 (0.02)	0.52 (0.05)	0.24 (0.02)
Cleanliness	0.78 (0.05)	0.44 (0.02)	0.75 (0.05)	0.36 (0.02)
Safe	0.88 (0.03)	0.64 (0.02)	0.74 (0.03)	0.46 (0.02)
Enough staff	0.72 (0.04)	0.39 (0.02)	-	-

**Table 7:** Goodness-of-Fit Indicators of a Three- Factor Confirmatory Model

Good-of Fit Statistic*	Combined		Ghana		Kenya	
	36-observed variables	20-observed variables	36-observed variables	20-observed variables	36-observed variables	20-observed variables
Chi-square, p-value	2150.827, p<0.001	694.801, p<0.001	2091.697, p<0.001	587.204, p<0.001	1343.446, p<0.001	417.207, p<0.001
Root mean squared error of approximation	0.048	0.054	0.067	0.069	0.050	0.055
Pclose	0.922	0.050	<0.001	<0.001	0.429	0.054
Comparative fit index	0.941	0.963	0.914	0.952	0.923	0.953
Tucker-Lewis index	0.903	0.931	0.859	0.912	0.873	0.913
Standardized root mean squared residual	0.046	0.041	0.055	0.041	0.045	0.040

* Model adopted is one with modification indices of a covariance of 10 or more

**Table 8:** Characteristics of scale items

	Internal Consistency reliability			Standardized scores			
	Number of items	Average interitem correlation	Scale reliability coefficient	Mean	Std. dev.	Min	Max
***Combined (N=1988)***							
Full 36-item scale	36	0.25	0.92	71.50	16.35	12.04	100
34-item scale*	34	0.28	0.93	71.13	17.26	10.78	100
20-item short scale	20	0.27	0.89	70.60	18.36	1.67	100
Dignity and Respect subscale	8	0.21	0.78	81.23	17.22	8.33	100
Communication and autonomy subscale	15	0.37	0.88	65.23	21.54	4.44	100
Responsive and supportive care subscale	13	0.22	0.78	72.75	15.16	15.38	100
Responsive and supportive care subscale*	11	0.30	0.82	71.81	17.55	0.00	100
***Kenya (N=996)***							
Full 36-item scale	36	0.22	0.91	68.84	15.78	21.30	100
34-item scale*	34	0.24	0.91	68.15	16.43	20.59	100
20-item short scale	20	0.27	0.88	67.27	17.53	15.00	100
Dignity and Respect subscale	8	0.29	0.77	79.69	17.33	20.83	100
Communication and autonomy subscale	15	0.28	0.86	60.36	21.00	13.33	100
Responsive and supportive care subscale	13	0.19	0.75	71.94	14.40	17.95	100
Responsive and supportive care subscale*	11	0.23	0.77	70.38	15.88	18.18	100
***Ghana (N=992)***							
Full 36-item scale	36	0.30	0.94	74.18	16.47	12.04	100
34-item scale*	34	0.33	0.94	74.11	17.56	10.78	100
20-item short scale	20	0.37	0.92	73.95	18.59	1.67	100
Dignity and Respect subscale	8	0.37	0.82	82.77	16.99	8.33	100
Communication and autonomy subscale	15	0.38	0.90	70.12	20.97	4.44	100
Responsive and supportive care subscale	13	0.30	0.85	73.56	15.85	15.38	100
Responsive and supportive care subscale*	11	0.37	0.87	73.26	18.98	0.00	100

The 34-item full scale and 11-item responsive and supportive care sub-scale exclude the two wait time variables

**Table 9:** Crosstabulation and Linear Regression on PCANC score, N=1988

	*Crosstab*			*Linear Regression on PCANC score*			
	N	Mean	SD	Coefficient	p-value	[95% conf. interval]
**Satisfaction with ANC**							
Dissatisfied	45	55.08	21.01	−15.34	0.00	−21.48	−9.21
Neither satisfied nor dissatisfied	100	52.57	13.32	−17.85	0.00	−20.58	−15.12
Satisfied	1360	70.42	15.39	Reference			
Very satisfied	483	79.98	13.50	9.55	0.00	8.10	11.01

**Perceived quality of ANC**							
Poor/Very Poor	36	45.81	15.03	−22.39	0.00	−27.34	−17.45
Fair	177	58.50	17.13	−9.71	0.00	−12.40	−7.01
Good	993	68.20	15.35	Reference			
Very good	616	78.04	12.15	9.84	0.00	8.48	11.19
Excellent	166	86.41	10.79	18.21	0.00	16.31	20.11

**Will return for ANC in future**							
No, never	187	69.01	17.07	−5.07	0.00	−7.63	−2.51
Yes, somewhat	304	60.32	16.95	−13.76	0.00	−15.81	−11.71
Yes, definitely	1497	74.08	15.09	Reference			

**Table 10:** All PCANC questions and final decisions

No	Label	Question	Decision for 36 item scale	Reason for inclusion/exclusion from 36 item scale	Decision for 20-item short scale
1	Greeted	Did they greet you well when you arrived or responded well if you greeted first?	Included	Adequate performance	Included
2	Treat you with respect	Did they treat you with respect?	Included	Adequate performance	Included
3	Friendly care	Were they friendly or nice to you?	Included	Adequate performance	Included
4	Knowledge valued	Did you feel your knowledge was valued? Did they appreciate or accept your ideas or knowledge about your health?	Included	Adequate performance	Excluded
5	Privacy not exposed	During physical exams (like abdominal and pelvic exams) were you covered up with a cloth or blanket or screened with a curtain so that you did not feel exposed?	Included	Adequate performance	Included
6	Info confidentiality	Did you feel your health information was kept confidential by the health workers?	Included	Adequate performance	Included
7	Auditory privacy	Did you feel you could discuss your problems with the health workers, without others not involved in your care overhearing your conversations?	Included	Adequate performance	Excluded
8	Family respected	Did the health workers respect your family or companions who were with you?	Excluded	Excluded because of high not applicable	Excluded
9	Verbal abuse	Did you feel they talked to you badly (For example, shouted at you, scolded, insulted, or threatened, you)?	Included	Moderate performance but retained for conceptual relevance	Excluded
10	Discrimination	Did you feel discriminated against by the health workers in any way? I.e., did you feel you were treated differently based on something about you?	Excluded	Excluded because of low loading	Excluded
11	Neglected	Did you feel the health workers avoided, ignored, or neglected you?	Excluded	Excluded because of low loading	Excluded
12	Introductions by provider	Did the health workers introduce themselves to you when they first saw you?	Included	Adequate performance	Included
13	Called preferred name	Did they call you by your name (or appropriately)?	Included	Adequate performance	Included
14	Heard and listened to	Did you feel heard by the health workers?	Included	Adequate performance	Included
15	Consent	Did the health workers ask your permission before examining or doing procedures on you?	Included	Moderate performance	Included
16	Involved in decisions	Did the health workers involve you in decisions about your care?	Included	Adequate performance	Included
17	Explain exams/procedures	Did they explain to you why they were doing examinations or tests on you?	Included	Adequate performance	Included
18	Understood tests	Did you understand the purpose of any examinations or tests you were asked to do? ... like urine or blood tests, ultrasound, etc., that you were asked to do including those you were asked to do outside the facility)?	Excluded	Excluded because of correlation with explain exams/procedures	Excluded
19	Told examination results	Did they tell you the results of examinations you did like your blood pressure and weight?	Included	Adequate performance	Excluded
29	Told test results	Did they tell you the results of tests you did like blood or urine tests?	Included	Adequate performance	Excluded
22	Explain medications	Did they explain to you why they were giving or prescribing you any medicine?	Included	Adequate performance	Included
21	Understood medicines	Did you understand the purpose of any medicines you were given or prescribed to go and buy?	Excluded	Excluded because of correlation with explain medicines	Excluded
23	Could ask any questions	Did you feel you could ask the health workers any questions you had?	Included	Adequate performance	Excluded
24	Encouraged questions	Did they encourage you to ask questions?	Included	Adequate performance	
25	Hold back questions	Did you hold back from asking questions for any reason?	Excluded	Excluded because of low loading	Excluded
26	Questions were answered	Do you feel your questions were answered when you asked them?	Included	Adequate performance	Excluded
27	Check understood information	Did they check that you understood information that was given to you?	Included	Adequate performance	Included
28	language they understood	Did the health workers speak to you in a language you could understand or using words you could understand?	Included	Adequate performance	Excluded
29	Asked about birth plan	Did a health worker discuss your birth plan with you (e.g., where you wanted to give birth or what you wanted during the birth?)	Included	Adequate performance	Included
30	Pressured	Did you feel forced into a decision by health workers?	Excluded	Excluded because of low loading	Excluded
31	Wait time for provider	How did you feel about the amount of time you had to wait to be seen by a health worker during antenatal visits (i.e., the time from when you arrived at the health facility to when you saw the midwife or doctor)?	Included	Included despite low loading because of conceptual relevance	Excluded
32	Wait time for labs or drugs	How did you feel about the amount of time it took you to get your labs done and/or get your drugs at the facility?	Included	Included despite low loading because of conceptual relevance	Excluded
33	Time with provider	How did you feel about the amount of time the doctor or midwife spent with you? (i.e., was it rushed or did they take their time with you)	Excluded	Excluded because of low loading	Excluded
34	Received best care	Did you feel they took the best care of you?	Included	Adequate performance	
35	Showed they cared	Did you feel the health workers cared about you?	Excluded	Excluded because of correlation with best care	Excluded
36	Physical wellbeing assessed	Did they ask you about your physical health?	Included	Adequate performance	Excluded
37	Mental/emotional wellbeing assessed	Did they ask you about your mental and emotional health?	Included	Adequate performance	Included
38	Resources for emotional/mental wellbeing	Did they give you the support to deal with your mental and emotional health?	Included	Adequate performance	Excluded
29	Attention when needed help	When you needed support from the providers with regards to your health, did they pay attention?	Included	Adequate performance	Excluded
40	Trust	Did you feel you could trust the health workers with regards to your care?	Included	Adequate performance	Included
41	Competence	Did you feel the health workers were competent?	Included	Adequate performance	Excluded
42	Washrooms	Could you use the washrooms in the facility if you needed to?	Included	Adequate performance	Included
43	Cleanliness	Did you feel the health facility environment, including the washrooms were clean?	Included	Adequate performance	Included
44	Safe	In general, did you feel safe (physically and psychologically) in the place you received antenatal care?	Included	Adequate performance	Included
45	Enough staff	Do you think there was enough health staff in the facility to care for you?	Included	Adequate performance	Excluded
46	Bribe	During your antenatal care, did any health worker at the facility ask you or your family for an unofficial payment?	Excluded	Excluded because of low loading	Excluded
47	Physical abuse	Did you feel they handled you badly (For example pushed, beat, slapped, pinched or physically restrained you)?	Excluded	Excluded because of low frequency and loading	Excluded
48	Language level they understood	Did they speak to you using words you could understand?	Excluded	Combined with language they understood	Excluded
49	Preferred clinic	Were you able to go to your preferred place/clinic for antenatal care?	Excluded	Excluded because of low relevance	Excluded
50	Preferred provider	Were you able to see your preferred provider for antenatal care?	Excluded	Excluded because of low relevance	Excluded

Notes: Last four not included in the CPIPE trial.

All response options except noted below are: 0, No, never | 1, Yes, a few times | 2, Yes, most of the time | 3, Yes, all the time |

Some include 4, N/a-Don’t know

9, 10, 11, 47: 0, No, never | 1, Yes, once | 2, Yes, a few times | 3, Yes, many times | 88, Refused to answer

1, 12, 28, 41: 0, No, none of them | 1, Yes, a few of them | 2, Yes, most of them | 3, Yes, all of them | 88, Refused to answer

31, 32: 0, It was just right | 1, It was somewhat long | 2, It was very long | 3, It was extremely long | 4, Not applicable | 88, Refused to answer

33: 0, It was just right | 1, It was too long | 2, It was somewhat short | 3, It was very short | 88, Refused to answer

## Data Availability

The datasets used and analyzed during the current study are available from the corresponding author [PAA] upon reasonable request.

## Abbreviations

PCANC: Person-centered antenatal care
LMICs: Low- and middle-income countries
US: United States
ANC: Antenatal care
SSA: sub-Saharan Africa
WHO: World Health Organization
PCMC: Person-centered maternity care
PCPC-US: Person-centered prenatal care scale
UER: Upper East Region
CHPS: Community-Based Health Planning and Services
UCSF: University of California, San Francisco
NHRC: Navrongo Health Research Center
EFA: Exploratory factor analysis
KMO: Kaiser-Meyer-Olkin
CFA: Confirmatory factor analysis
RMSEA: Root mean square error of approximation
p of close fit: pclose
CFI: Comparative fit index
TLI: Tucker-Lewis index
SRMR: Standardized root mean squared residual
NA: Not applicable
